# How is the Beginning and End of Frequent Laughter Associated With Changes in Loneliness Amongst Older People in Japan? A JAGES Longitudinal Study

**DOI:** 10.1002/gps.70177

**Published:** 2025-11-20

**Authors:** André Hajek, Naoki Kondo, Hans‐Helmut König

**Affiliations:** ^1^ Department of Health Economics and Health Services Research University Medical Center Hamburg‐Eppendorf Hamburg Center for Health Economics Hamburg Germany; ^2^ Department of Social Epidemiology Graduate School of Medicine and School of Public Health Kyoto University Kyoto Japan

**Keywords:** asymmetric effects, JAGES, laughing, loneliness, social exclusion, social isolation, social withdrawal

## Abstract

**Objective:**

Little is known about the association between frequency of laughter and loneliness—in particular based on longitudinal data. Therefore, our aim was to examine how the onset and the end of frequent laughter is associated with changes in loneliness amongst older people in Japan.

**Methods/Design:**

Longitudinal data were taken from the Japan Gerontological Evaluation Study (JAGES, waves 6 and 7 with *n* = 5262 observations, mean age was 74.4 years, SD: 5.8 years). The widely used and psychometrically sound UCLA‐3 was used to quantify loneliness. Frequency of laughing served as key independent variable. Asymmetric linear fixed effects regressions were used, adjusting for several time‐varying covariates.

**Results:**

After adjusting for several sociodemographic, lifestyle‐related and health‐related time‐varying factors, there was a significant association between the onset of frequent laughing and decreases in loneliness (*β* = −0.18, *p* < 0.05). Age and sex did not moderate this association. In contrast, the cessation of frequent laughter was not significantly associated with changes in loneliness.

**Conclusions:**

Starting frequent laughter may help to avoid negative consequences such as loneliness among older adults in Japan. This may emphasize the importance of finding ways to start laughing frequently. Efforts to reduce loneliness are important because it is in turn associated with outcomes such as morbidity and mortality.

## Key Points

- Longitudinal data were taken from the Japan Gerontological Evaluation Study.
- Onset of frequent laughing was associated with decreases in loneliness.
- Cessation of frequent laughter was not significantly associated with loneliness.
- Starting frequent laughter may help to avoid negative consequences.
- This stresses the importance of finding ways to start laughing frequently.

## 1 Introduction

Following Gonot‐Schoupinsky et al. [1], “laughter is predominantly a physical behavior, occurring alone or socially. It is often used as a form of verbal expression or communication” (4–5). Laughter is often accompanied by changes in facial expression and sounds of laughing. Previous research revealed that while some people laugh nearly daily, other rarely laugh at all. For instance, one study revealed that about 27% of individuals aged 20 years and over laughed nearly daily, whereas nearly 13% of the participants laughed “almost never” based on data from the Fukushima Health Management Survey [2]. A recent German study found that more than one out of three individuals reported daily laughter, whereas only about one out of 10 individuals reported laughing almost never [3].

Several former studies have shown that laughing frequency is of relevance for health outcomes including functional impairment [4], mental health [5] and frailty [6]. It is also associated with all‐cause mortality [7]. The beneficial impact of laughter is largely linked to various factors, including better immune function and decreased inflammation [8, 9] which come from the positive emotions caused by laughing. The frequency of laughter can also buffer stress [10]. Beyond the positive health effects, frequency of laughter might be associated with other factors such as loneliness—a negative emotion reflecting a qualitative or quantitative difference between actual and desired social relationships [11].

A previous study showed an association between insufficient social relationships and living alone with a reduced laughter frequency using cross‐sectional data from the Japan Gerontological Evaluation Study (JAGES). Using data from the Fukushima Health Management Survey (which includes adults aged 20 years and over), another study showed an association between more family members and an increased laughing frequency [2]. In a further study, a higher frequency of social participation was associated with a higher frequency of laughter among older adults in Japan [12]. Similar results were also found by Nagai et al. [13].

Intervention studies on the impact of laughter therapy on loneliness have yielded mixed results. A pilot study (non‐randomized; small sample) [14] suggested that laughter therapy may reduce loneliness in older adults residing in nursing homes in Turkey, while a recent randomized controlled trial (with a small sample) from Turkey found no effect of online laughter therapy on nursing students' loneliness during the COVID‐19 pandemic [15]. Similarly, another study found no impact on loneliness of healthy employees in India [16], whereas a laughter yoga program did show positive effects for older adults in a Turkish nursing home [17]. Due to the limited knowledge in this research area, this longitudinal study aimed to examine how the onset and the end of frequent laughter is associated with changes in loneliness amongst older people in Japan.

This study extends the existing literature by focusing explicitly on the link between frequent laughter and loneliness *based on a longitudinal approach*. Panel econometric techniques were used to mitigate the key challenge of unobserved heterogeneity—a key challenge when dealing with observational data. As the very first study, we examine whether the association of the *onset* and *cessation* of frequent laughter with loneliness differs (i.e., whether asymmetric effects exist). In contrast to the limited interventional studies that focus on specific therapeutic modalities (such as laughter yoga), our focus is on laughter in everyday life, which is crucial to ensure ecological validity.

Several factors might explain the link between frequency of laughter and loneliness. e.g., sharing laughter can strengthen emotional bonds [18], such as during conversations with friends and family. Laughter also triggers the release of endorphins, which can improve well‐being and decrease stress. This may promote a sense of social bonding [19]. Laughing can also help to reduce inhibitions and anxiety, encouraging individuals to be more open and sociable [3]. In contrast, inappropriate laughter (e.g., laughing at someone's else's expense) can be perceived as insensitive or offensive, potentially alienating others. This could lead to the withdrawal of friends and contribute to loneliness [3]. However, we assume that this is rarely the case ‐ especially in a country like Japan that strives for harmony. We assume that the positive outcomes of laughter clearly outweigh the negatives.

Moreover, we expect asymmetric effects of frequent laughter: in particular, while the cessation of frequent laughter is likely to lead to a marked increase in loneliness, the onset of frequent laughter might lead to only a small reduction in loneliness. In this context, negative experiences might have a greater overall contribution to loneliness than positive ones [20]. Potential implications may be that ensuring regular laughter may help mitigate adverse outcomes in terms of loneliness. Addressing loneliness is important because it is associated with several negative health outcomes, including increased morbidity and mortality [21, 22].

## 2 Materials and Methods

### 2.1 Sample

Longitudinal data were used from the prospective cohort study JAGES. It includes community‐dwelling Japanese residents aged 65 years and over not being physically or cognitively disabled and who are not eligible for long‐term healthcare insurance benefits at the time of the baseline survey. Various topics are included such as psychological factors, health factors and social determinants of health.

The first wave took place in 2002–2003. Follow‐up waves took place every three years. For data availability reasons (i.e., the key variables of laughter and loneliness were not consistently surveyed in other waves), we used data from wave 6 (December 2019 and January 2020) and wave 7 (November to December 2022). Since wave 3, the survey includes a wide area in Japan. Mostly self‐administered questionnaires were used in the JAGES. Given the fact that older adults are interviewed and also compared to European surveys [23, 24], the response rates of the JAGES are high. For example, the response rates in wave 6 and 7 were slightly less than 70%. Further details regarding JAGES is available elsewhere [25].

Permission to use the JAGES data was obtained by the JAGES investigators. Informed consent was provided by the participants. The Ethics Committee on the Research of Human Subjects at Nihon Fukushi University (no. 10–05) approved the JAGES protocol.

### 2.2 Loneliness

Loneliness was quantified using the UCLA Loneliness Scale (UCLA‐3), consisting of three items. It is a widely used psychometrically sound tool [26], consisting of the following items: “How often do you feel that you lack companionship?”; “How often do you feel left out?“; „How often do you feel isolated from others?”. Each item could be answered on a three‐point scale: 1 = “hardly ever”; 2 = ”some of the time”; 3 = ”often” [27]. The scores for each question were summed to a loneliness score ranging from 3 to 9, whereby higher values correspond to higher loneliness levels.

### 2.3 Key Independent Variables

Based on previous research [28, 29], the frequency of laughter was measured based on the self‐reported frequency of laughing out loud (four categories: 1 = almost every day, 2 = one to five times a week, 3 = one to three times a month, 4 = almost never). Laughing out loud “almost every day” was defined as frequent laughter in our main model (coded as one; otherwise coded as 0), following previous research [13]. Participants whose code changed from 0 to 1 from wave 6 to wave 7 were classified as having started (or begun) experiencing frequent laughter. Conversely, those whose code changed from 1 to 0 during this period were classified as having ceased (or ended) frequent laughter. In a robustness check, laughing out loud at least “one to five times per week” was defined as frequent laughter.

### 2.4 Time‐Varying Covariates

Time‐varying covariates were selected based on previous research [30, 31, 32] including sociodemographic, lifestyle‐related and health‐related factors. Time‐varying sociodemographic factors include age (in years) and marital status (1 = spouse including common‐law marriage, 2 = widowed, 3 = divorced, 4 = never married, 5 = other). Time‐varying lifestyle‐related factors include cigarette smoking behavior (including heated cigarettes/electronic cigarettes), alcohol consumption, fruit/vegetable consumption, frequency of meeting with friends and acquaintances, and walking behavior. The following response options were available for the smoke variable: 1 = smokes almost every day, 2 = smokes occasionally, 3 = quit smoking within 5 years and don't smoke now, 4 = quit smoking more than 5 years ago and do not smoke now, 5 = never smoked. Alcohol consumption was also quantified using four response categories (1 = currently taking it, 2 = stopped taking it within 5 years and not taking it now, 3 = stopped drinking more than 5 years ago and do not drink now, 4 = never). Fruit/vegetable consumption in the past month was measured (1 = at least twice daily, 2 = once daily, 3 = 4–6 times a week, 4 = 2–3 times a week, 5 = once a week, 6 = less than once a week, 7 = not eaten). The frequency of meeting with friends and acquaintances was also used (1 = 4 times a week or more, 2 = 2 to 3 times a week, 3 = 1 time a week, 4 = 1–3 times a month, 5 = a few times a year, 6 = not seen). Walking behavior was quantified using the question: “On average, how many minutes do you walk per day in total?” (1 = less than 30 min, 2 = 30–59 min, 3 = 60–89 min, 4 = 90 min or more). As regards to health‐related covariates, self‐rated health, functional impairment, depressive symptoms and a count score for chronic conditions was used. A single‐item tool was used to quantify self‐rated health (1 = excellent, 2 = good, 3 = fair, 4 = poor). Functional impairment was measured using the question “Do you have any health problems that affect your daily life? (e.g., effects on daily activities such as getting up, dressing, eating, bathing, going out, work, schoolwork, exercise, etc.)” (1 = yes, 2 = no). Depressive symptoms were measured using the Geriatric Depression Scale (15‐item version). The final score ranges from 0 to 15, with higher values reflecting more depressive symptoms. The Japanese version of the GDS‐15 has excellent psychometric characteristics [33]. Our count of chronic conditions include 17 chronic conditions (hypertension; stroke (cerebral hemorrhage, cerebral infarction, etc.); heart disease; diabetes mellitus; hyperlipidemia (dyslipidemia); respiratory disease (pneumonia, bronchitis, etc.); diseases of the gastrointestinal tract, liver, and gall bladder; diseases of kidney and prostate; musculoskeletal diseases (osteoporosis, hip arthritis, etc.); trauma (falls, fractures, etc.); cancer (malignant neoplasms); blood and immune diseases; dementia (Alzheimer's disease, etc.); Parkinson's disease; eye disease; ear disease; other). The (nearly) time‐constant factor education (years of school education: 1 = less than 6 years, 2 = 6–9 years, 3 = 10–12 years, 4 = more than 13 years, 5 = other) and sex (men; women) were used for descriptive purposes (education) or to test potential moderating effects (sex).

### 2.5 Statistics

First, descriptive statistics were calculated (means with standard deviations or counts with percentages) to characterize the analytic sample. Then, asymmetric linear fixed effects (FE) regressions [34] were performed to examine the relationship between laughing frequency and loneliness longitudinally. These FE models were adjusted for time‐varying factors mentioned in the previous section. More precisely, first unadjusted models are shown. Then, we adjusted for time‐varying sociodemographic covariates. Subsequently, time‐varying lifestyle covariates were added. In the main model, time‐varying health‐related covariates were added. In an exploratory fashion, it was tested whether sex and age moderate the associations of interest (by including respective interaction terms). We also tested whether our results varied according to the definition of frequent laughter.

In general, a key advantage of FE regressions is their ability to provide consistent estimates under weak assumptions. Specifically, they yield consistent estimates even when unobserved or observed time‐invariant factors are systematically associated with the explanatory variables; unlike random‐effects regressions, which would produce inconsistent estimates under such conditions [35].

Generally, FE regressions focus on changes within individuals over time (e.g., shifts in laughing frequency between JAGES waves 6 and 7). This allows an analysis of how the onset and cessation of frequent laughter is associated with intraindividual changes in loneliness from wave 6 to 7. Thus, only time‐varying factors can be included in FE regressions as explanatory variables. Conversely, this means that time‐invariant factors such as sex—cannot be included as main effects in FE regressions, but can be used as moderating factors, as in our current study. Both observed and unobserved time‐invariant variables are inherently controlled in a FE framework. This focus on individuals experiencing changes in both independent and dependent variables is not a shortcoming of the FE approach. It reflects the fact that only a subset of the population experiences such changes over time.

Traditional FE models typically assume symmetric effects—the association between the onset of frequent laughter and loneliness is the same in magnitude as that of its end in absolute terms. However, we expect that asymmetric effects are more plausible since negative events—end of frequent laughter—are often more influential than positive events (the start of frequent laughter) [20]. Therefore, we employed asymmetric linear FE regressions in our analysis. Following Stock and Watson, we also calculated cluster‐robust standard errors (clustering errors at the individual level) [36].

McDonald's omega was assessed using a tool developed by Shaw [37]. A *p*‐value of less than 0.05 was considered statistically significant. All statistical analyses were carried out using StataNow 18.5 (Stata Corp., College Station, Texas).

## 3 Results

### 3.1 Sample Characteristics

Sample characteristics of the analytic sample are presented in Table 1. Overall, 2530 respondents (48.1%) were female and the average age was 74.4 years (SD: 5.8 years; ranging from 65 to 97 years). The average loneliness score was 4.0 (SD: 1.4). In our study, Cronbach's alpha for the UCLA‐3 was 0.80 in wave 6 and 0.81 in wave 7. McDonald's omega was 0.83 in both waves. In total, 2429 respondents (46.3%) had 10–12 years of school education. More details are shown in Table 1. In the analytic sample, 243 individuals started and 306 individuals stopped laughing frequently during the observation period (i.e., from wave 6 to wave 7).

**TABLE 1:** Sample characteristics (total sample, pooled over both waves, *n* = 5262 observations).

Variables	Mean (SD)/*n* (%)
Sex: *n* (%)	
Men	2732 (51.9)
Women	2530 (48.1)
Age: Mean (SD)	74.4 (5.8)
Years of school education: *n* (%)	
Less than 6 years	24 (0.5)
6–9 years	1116 (21.3)
10–12 years	2429 (46.3)
More than 13 years	1632 (31.1)
Other	42 (0.8)
Marital status: *n* (%)	
Spouse including common‐law marriage	4017 (76.3)
Widowed	869 (16.5)
Divorced	216 (4.1)
Never married	138 (2.6)
Other	22 (0.4)
Smoking behavior: *n* (%)	
Smokes almost every day	431 (8.2)
Smokes occasionally	64 (1.2)
Quit smoking within 5 years and don't smoke now	153 (2.9)
Quit smoking more than 5 years ago and do not smoke now	1611 (30.6)
Never smoked	3003 (57.1)
Alcohol consumption: *n* (%)	
Currently taking it	2263 (43.0)
Stopped taking it within 5 years and not taking it now	242 (4.6)
Stopped drinking more than 5 years ago and do not drink now	384 (7.3)
Never	2373 (45.1)
Fruit/vegetable consumption: *n* (%)	
At least twice daily	2387 (45.4)
Once daily	1925 (36.6)
4–6 times a week	567 (10.8)
2–3 times a week	308 (5.9)
Once a week	50 (1.0)
Less than once a week	21 (0.4)
Not eaten	4 (0.1)
Frequency of meeting with friends and acquaintances: *n* (%)	
4 times a week or more	761 (14.5)
2 to 3 times a week	1056 (20.1)
1 time a week	681 (12.9)
1–3 times a month	1168 (22.2)
A few times a year	1105 (21.0)
Not seen	491 (9.3)
Walking behavior (minutes per day): *n* (%)	
Less than 30 min	1381 (26.2)
30–59 min	1967 (37.4)
60–89 min	927 (17.6)
90 min	987 (18.8)
Self‐rated health: *n* (%)	
Excellent	795 (15.1)
Good	3776 (71.8)
Fair	635 (12.1)
Poor	56 (1.1)
Functional impairment: *n* (%)	
Yes	408 (7.8)
No	4854 (92.2)
Depressive symptoms (GDS‐15, 0 to 15, with higher values reflecting more depressive symptoms): Mean (SD)	2.9 (2.9)
Count of chronic conditions (0–17, with higher values reflecting more chronic conditions): Mean (SD)	1.6 (1.2)
Loneliness (UCLA‐3, 3 to 9, with higher values reflecting higher loneliness levels): Mean (SD)	4.0 (1.4)

### 3.2 Regression Analysis

In Table 2, findings of both unadjusted and adjusted asymmetric linear FE regressions are displayed. In the unadjusted model, the onset of frequent laughter was not significantly associated with changes in loneliness. In contrast, stopping laughing frequently was significantly associated with increases in loneliness scores (*β* = 0.37, *p* < 0.001).

**TABLE 2:** Frequent laughter (laughing almost every day) and loneliness. Findings of asymmetric linear FE regressions.

Independent variables	Loneliness			
Onset of frequent laughter	0.11+	−0.23***	−0.23**	−0.18*
	(−0.02–0.24)	(−0.36 to −0.09)	(−0.38 to −0.09)	(−0.34 to −0.01)
End of frequent laughter	0.37***	0.05	0.01	−0.06
	(0.27–0.47)	(−0.06–0.16)	(−0.11–0.12)	(−0.19–0.08)
Sociodemographic time‐varying covariates		✓	✓	✓
Lifestyle‐related time‐varying covariates			✓	✓
Health‐related time‐varying covariates				✓
Constant	3.97***	−4.38***	−4.36***	−4.50***
	(3.96–3.98)	(−5.48 to −3.28)	(−5.57 to −3.15)	(−5.93 to −3.07)
Observations	8518	8362	7242	5262
Number of individuals	4259	4181	3621	2631
*R* ^2^	0.01	0.07	0.07	0.11

*Note:* Unstandardized beta‐coefficients; 95% confidence intervals in parentheses. Sociodemographic time‐varying covariates: age and marital status; lifestyle‐related time‐varying covariates: cigarette smoking behavior, alcohol consumption, fruit/vegetable consumption, frequency of meeting with friends and acquaintances, and walking behavior; health‐related time‐varying covariates: self‐rated health, functional impairment, depressive symptoms, and count score for chronic conditions.

^*^ *p* < 0.05.

^**^ *p* < 0.01.

^***^ *p* < 0.001.

^+^ *p* < 0.10.

In all adjusted regression models, the significant association between stopping laughing frequently disappeared. However, the association between the onset of frequent laughter and decreases in loneliness gained statistical significance in all adjusted models (e.g., in the fully adjusted model: *β* = −0.18, *p* < 0.05).

In a robustness check, frequent laughter was defined as laughing out loud at least one to five times per week. These findings can be found in Table 3 in detail. Overall, such findings are similar (in terms of significance and effect size) compared to our main analyses presented in Table 2. In the fully adjusted model, there was a significant association between the onset of frequent laughing and decreases in loneliness (*β* = −0.23, *p* < 0.05), whereas the cessation of frequent laughter was not significantly associated with changes in loneliness in this model.

**TABLE 3:** Frequent laughter (at least one to five times a week) and loneliness. Findings of asymmetric linear FE regressions.

Independent variables	Loneliness			
Onset of frequent laughter	0.04	−0.28***	−0.25**	−0.23*
	(−0.12–0.20)	(−0.44 to −0.11)	(−0.42 to −0.07)	(−0.44 to −0.02)
End of frequent laughter	0.52***	0.22**	0.15+	0.07
	(0.37–0.68)	(0.07–0.38)	(−0.01–0.32)	(−0.12–0.25)
Sociodemographic time‐varying covariates		✓	✓	✓
Lifestyle‐related time‐varying covariates			✓	✓
Health‐related time‐varying covariates				✓
Constant	3.97***	−3.98***	−3.90***	−4.14***
	(3.96–3.98)	(−5.01 to −2.96)	(−5.06 to −2.74)	(−5.50 to −2.78)
Observations	8518	8362	7242	5262
Number of individuals	4259	4181	3621	2631
*R* ^2^	0.01	0.07	0.08	0.11

*Note:* Unstandardized beta‐coefficients; 95% confidence intervals in parentheses. Sociodemographic time‐varying covariates: age and marital status; lifestyle‐related time‐varying covariates: cigarette smoking behavior, alcohol consumption, fruit/vegetable consumption, frequency of meeting with friends and acquaintances, and walking behavior; health‐related time‐varying covariates: self‐rated health, functional impairment, depressive symptoms, and count score for chronic conditions.

^*^ *p* < 0.05.

^**^ *p* < 0.01.

^***^ *p* < 0.001.

^+^ *p* < 0.10.

We also tested whether sex or age moderates the association of the onset and end of frequent laughter with changes in loneliness by including respective interaction terms (e.g., sex x onset of frequent laughter; sex x end of frequent laughter etc.). However, none of the interaction terms achieved statistical significance (sex x onset of frequent laughter: *β* = −0.23, *p* = 0.15; sex x end of frequent laughter: *β* = −0.12, *p* = 0.33; age x onset of frequent laughter: *β* = 0.02, *p* = 0.17; age x end of frequent laughter: *β* = −0.02, *p* = 0.16).

## 4 Discussion

This present study aimed to investigate how the onset and the end of frequent laughter is associated with changes in loneliness among older adults in Japan based on data from JAGES. Our key findings were as follows: Adjusted asymmetric linear FE regressions showed that there was a robust association between the onset of laughing frequently and decreases in loneliness. Age and sex did not moderate this association. This indicates that such associations were similar across sociodemographic groups. The cessation of frequent laughter was not significantly associated with changes in loneliness. This is the first study investigating how the onset and the end of frequent laughter is associated with changes in loneliness in general. Thus, this current study extends our current understanding of the link between laughing frequently and loneliness. A previous non‐randomized pilot study showed that loneliness decreased among nursing home residents after laughter therapy in the intervention group [14]. Other research showed a positive link between social participation and laughter frequency among older adults in Japan based on observational data [12, 13].

Our initial expectations were that the cessation of frequent laughter might lead to a marked increase in loneliness, whereas the onset of frequent laughter might lead to only a small reduction in loneliness. However, our assumptions are only partially confirmed by the adjusted results. Potential factors explaining the link between the onset of frequent laughter and decreases in loneliness may be that frequent laughter is associated with the ability to improve well‐being, enhance emotional connections, alleviate stress, and diminish fear [19]. One possible role of starting to laugh frequently is to strengthen emotional bonds, facilitating better social integration. In contrast, stopping to laugh frequently, might contribute less to existing relationships. While stopping to laugh frequently might be linked to dissatisfaction or stress levels, this might not directly affect loneliness levels. Furthermore, starting to laugh frequently might confirm belonging to a group which in turn might reduce loneliness levels. In contrast, cessation, might be perceived as an individual choice that does not inevitably affect existing social relationships. Moreover, feedback loops might play a role here: Individuals beginning to laugh frequently might be perceived positively. This might favor social relationships. In contrast, when individuals stop to laugh frequently, their social relationships might not necessarily suffer, for example, it might be the case that friends and acquaintances continue to spend time with them. Thus, loneliness might not necessarily increase. However, it should be emphasized that these are potential explanations which should be tested in upcoming research.

Some strengths and limitations of the current study are worth describing. This is the first study exploring how the onset and the end of frequent laughter is associated with changes in loneliness amongst older people in Japan. Longitudinal data of the JAGES study were used. The challenge of unobserved heterogeneity was addressed using a FE approach. The psychometrically sound UCLA‐3 was used to quantify loneliness. However, we recommend future research based on tools such as the De Jong Gierveld tool to distinguish between emotional and social loneliness [38]. The self‐reported frequency of laughter served as a key independent factor. The use of such a one‐item tool may introduce measurement error. Therefore, we recommend upcoming research that deals in depth with the frequency of laughter (e.g., based on objective methods; taking into account the exact occasions of laughter, e.g., at a get‐together with friends and family or when watching videos on the internet alone).

In conclusion, starting frequent laughter may help to avoid negative consequences such as loneliness. Efforts to decrease loneliness are relevant since loneliness in turn contributes to morbidity and mortality. This stresses the importance of identifying ways to laugh often. Future research should examine the link between frequent laughter and loneliness in different settings (e.g., among individuals residing in nursing homes) and countries. We also recommend qualitative research to get a better understanding of this association. The situation of laughter should also be examined more closely in this context. Furthermore, frequent laughter can possibly also act as an ice‐breaker to intensify social contacts, which then remain even if individuals no longer laugh frequently. This should be investigated in upcoming research.

## Author Contributions

**André Hajek:** conceptualization, data curation, methodology, project administration, visualization, roles/writing – original draft, writing – review and editing, formal analysis. **Naoki Kondo:** conceptualization, writing – review and editing, visualization. **Hans‐Helmut König:** conceptualization, resources, writing – review and editing, supervision, visualization.

## Funding

This study used data from JAGES (the Japan Gerontological Evaluation Study). This study was supported by Grant‐in‐Aid for Scientific Research (19K02200, 20H00557, 20H03954, 20K02176, 20K10540, 20K13721, 20K19534, 21H03153, 21H03196, 21K02001, 21K10323, 21K11108, 21K17302, 21K17308, 21K17322, 22H00934, 22H03299, 22J00662, 22J01409, 22K04450, 22K10564, 22K11101, 22K13558, 22K17265, 22K17409, 23K16320, 23H00449, 23H03117 from JSPS (Japan Society for the Promotion of Science), Health Labor Sciences Research Grants (19FA1012, 19FA2001, 21FA1012, 22FA2001, 22FA1010, 22FG2001), the Research Funding for Longevity Sciences from National Center for Geriatrics and Gerontology (21–20), Research Institute of Science and Technology for Society (JPMJOP1831) from the Japan Science and Technology (JST), a grant from Japan Health Promotion & Fitness Foundation, contribution by Department of Active Aging, Niigata University Graduate School of Medical and Dental Sciences (donated by Tokamachi city, Niigata), TMDU priority research areas grant and National Research Institute for Earth Science and Disaster Resilience. The views and opinions expressed in this article are those of the authors and do not necessarily reflect the official policy or position of the respective funding organizations.

## Conflicts of Interest

The authors declare no conflicts of interest.

## Data Availability

The data underlying this study is from the JAGES and contain sensitive information. Data for research purposes is available upon request. Requests for the JAGES data can be made to e‐mail: dataadmin.ml@jages.net.
